# Dietary Fermentation with *Lactobacillus* sp. and *Bacillus* sp. Modulates Rumen Transcriptomic and Microbiota Profiles in *Bos taurus*

**DOI:** 10.3390/ijms26146816

**Published:** 2025-07-16

**Authors:** Jeong Sung Jung, Dahye Kim, Anand Singaravelu, Ilavenil Soundharrajan, Ki Choon Choi

**Affiliations:** 1 Forage Production System Division, National Institute of Animal Science, RDA, Cheonan 31000, Republic of Korea; jjs3873@korea.kr; 2 Animal Genomics and Bioinformatics Division, National Institute of Animal Science, Rural Development Administration (RDA), Wanju 55365, Republic of Korea; dhkim0724@korea.kr; 3 Department of Chemistry, Saveetha Engineering College, Thandalam 602105, Tamil Nadu, India; anands@saveetha.ac.in

**Keywords:** *Lactobacillus* sp., *Bacillus* sp., fermented feed, *Bos-taurus*, transcriptome, microbial dynamics

## Abstract

Animal feed made from fermented agricultural residues using *Lactobacillus* sp. *and Bacillus* sp. has received significant attention. However, interactions between differentially expressed genes (DEGs) in adipose, liver, and muscle tissues and bacteria or fungi in the rumen remain largely unknown. This study determined effects of normal diet feed (NF) and alternative diet feed made by *Lactobacillus* sp. *and Bacillus* sp. (AF) on gene expression in major metabolic organs and on microbial populations in the rumen of *Bos-Taurus* using high-throughput sequencing methods. Rumen bacteria/fungi interaction with DEGs in key metabolic organs was also investigated. According to our findings, 34, 36, and 28 genes were differentially expressed in adipose, liver, and muscle tissues, respectively. Most DEGs were associated with osteoclast differentiation and immune functions. Microbial dynamics analysis showed that the AF diet significantly (*p* < 0.05) increased *Firmicutes* but reduced *Bacterioidetes* abundances. At the genus level, *Faecalicatena*, *Intestinimonas*, *Lachnoclostridium*, *Faecalicatena*, and *Intestinimonas* were significantly higher (*p* < 0.05) in animals fed with the AF diet. Regarding fungal populations, *Neocallimastigomycota* accounted for 98.2% in the NF diet and 86.88% in the AF diet. AF feeding increased *Orpinomyces* and *Piromyces* but decreased *Neocallimastix* abundances. These findings highlight the potential of fermented feeds to improve metabolic responses and rumen microbial balance, contributing to enhanced animal performance.

## 1. Introduction

It is a key priority for the livestock industry to improve the efficiency and sustainability of ruminant production systems, especially given the limited feed resources and rising feed costs. A promising strategy for improving feed utilization and animal health is fermentation of agricultural byproducts into value-added feeds [1,2]. During fermentation processes, lactic acid bacteria (*Lactobacillus* spp.) and spore-forming bacteria (*Bacillus* spp.) degrade complex polysaccharides and produce organic acids, enzymes, and other bioactive compounds that can improve ruminant nutrient availability and gut microbe balance [3,4]. Livestock digestion and nutrient absorption can be improved by substituting fermented feed made from agricultural crops byproducts with grass and legumes rather than commercial concentrates. Furthermore, fermented feed contains lactic acid, acetic acid, and other essential organic acids that can reduce pathogenic microbial populations in cattle’s rumens [5]. Fermented feed also contains significant numbers of beneficial microbes that can regulate gut microbial dynamics. Use of fermented feed can significantly reduce costs because it can maximize feed efficiency and reduce feces production due to high nutrient digestion and absorption [5,6]. Fermentation can also be used to kill residual pathogenic microorganisms in feed [7] and improves digestion, nutrient absorption, the immune system, and overall health [8,9]. Fermented feeds may also reduce anti-nutritional factors and enhance digestibility of fibrous crop residues, making them nutritionally valuable feeds for animals [10]. However, the molecular mechanisms behind the improvement in performance of ruminants from fermented feeds are still poorly understood. Several studies have investigated feed-induced changes in rumen microbial ecology [11,12]. Still, little is known about the transcriptional changes in metabolically important tissues like livers, muscles, and adipose tissues. The gene expression profiles of these tissues reflect how animals respond to different nutritional regimens, such as energy metabolism, immunity, and nutrient partitioning. The identification of metabolic pathways that contribute to enhanced feed conversion efficiency and growth in ruminants requires an understanding of these interactions.

The rumen microbiome is extremely complex, containing bacteria, archaea, fungi, viruses, and protozoa [13,14]. Microorganisms in the rumen can degrade complex feeds into volatile fatty acids (VFA) and ammonia. They can also synthesize vitamin B and microbial cell proteins essential for animal health [15]. Several factors, including age, diet, health status, host species, geographical location, and antibiotic treatment, can influence the structure of the ruminal microbial community [16,17,18]. The high-grain diet could change the microbial dynamics of the rumen and the major fermentation products in a significant way [18,19]. An animal diet from forage is dominated by cellulolytic and fibrolytic bacteria known to be degraded cellulose and hemicellulose, whereas a concentrate diet is dominated by amylolytic bacteria known to ferment sugars and starch. A strong correlation between feed efficiency and the structure of the ruminal microbial community has been shown [20]. Microbes in the rumen could promote efficiency of feed and average daily gain in animals [21,22]. Genetic networks are based on the concept that genes and their products can interact through complex relationships, with changes in the behavior of one gene propagating to other genes [23,24]. A transcriptomic analysis reveals a more complex transcriptional regulation than differentially expressed genes (DEGs) because co-expressed genes orchestrate complex traits. The technique can also be used to identify hub genes, since hub genes are expected to control the expression of dozens of other genes within a module [25,26]. This present study addresses this knowledge gap by evaluating the effects of fermented agricultural byproduct-based feed on animal performance. Possible mechanisms of alternative diet supplementation on the adipose, liver, and muscle tissue transcriptomes and rumen microbial dynamics of the *Bos taurus* rumen are elucidated. In addition, profile shifts in bacterial and fungal populations in rumen are explored, as well as correlations between microbial taxa and tissue-specific DEGs. Our findings will contribute to a deeper understanding of diet–microbiome–host interactions and will help us optimize fermented feeds for improved rumen health and animal performance.

## 2. Results

### 2.1. Animal Growth and Performance by Diet Variations

A preliminary study analyzing the impacts of alternative diet supplementation on Hanwoo steer growth performance and meat quality was previously published [6]. Dry matter intake, total body weights, average daily gain, and carcass production were significantly increased in Hanwoo steers fed fermented feed (Alternative diet feed, AF) as compared to Hanwoo steers fed normal diet feed (NF). Feed conversion ratios (FCR) were also significantly higher in Hanwoo steers fed an AF diet during both early and late fattening periods compared to those fed NF. Animals fed AF showed significantly increased final body weight compared to NF group animals (*p* < 0.05) for the total period. Other parameters, such as ADG, feed intake, and FCR were not significantly different between NF and AF-fed animals.

### 2.2. RNA Sequence Alignment and Differentially Expressed Genes (DEGs)

**Figure 1:**
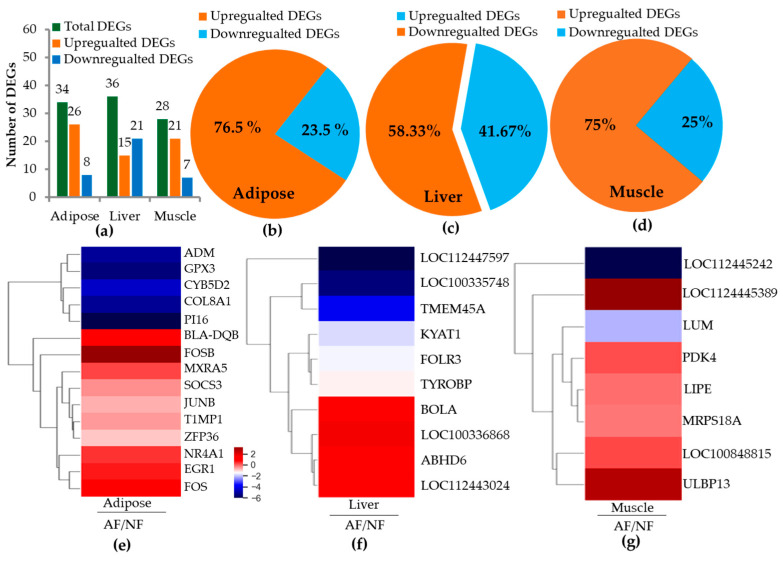
Differentially expressed genes in adipose, liver, and muscle tissues of cattle by diet variations. The animal was fed a normal-feed (NF) diet and an alternative-feed diet (AF) until the patenting stage. Transcriptome analysis was performed on adipose, liver, and muscle tissues. Total DEG numbers in major metabolic organs (**a**), and the percentage of DEGs expressed in adipose (**b**), liver (**c**), and muscle (**d**) are represented by Venn diagram. A heat map was generated using ExDEGA graphicPlus software-5.0.0. to correlate DEGs in adipose (**e**), liver (**f**), and muscle (**g**) between diet variations. The heat map correlation was determined by considering two-fold changes in DEGs at a significant level of 0.05. NF: Normal diet feed, AF alternative diet feed.

Transcriptome analysis was successfully completed for all experimental tissues (average: 26.14 and 24.44 million for adipose tissue, 24.1 and 23.1 million for liver tissue, and 26.0 and 26.0 million for muscle from NF and AF, respectively). To identify DEGs in experimental tissues, fold changes > 1.5 with *p*-value < 0.05 were set as selection criteria. Figure 1a–d show total numbers of differentially expressed genes (DEGs) in adipose, liver, and muscle tissues of *Bos taurus*. A total of 34 genes in adipose tissues, 36 in liver tissues, and 28 genes in muscle tissues of *Bos taurus* were identified as differentially expressed between NF- and AF-fed groups (Table 1, Table 2 and Table 3). Among identified DEGs in different tissues, 26 (76.5%), 15 (58.33%), and 21 (75%) genes were upregulated in adipose, liver, and muscle tissues, respectively. The total number of genes significantly (*p* < 0.05) downregulated in adipose, liver, and muscle tissues of *Bos taurus* in the AF group compared to the NF group were eight (23.5%), 21 (41.6%), and seven (25%), respectively. Figure 1e–g provides visual demonstration of a heatmap correlation pertaining to DEGs from *Bos taurus* adipose, liver, and muscle tissues, highlighting the most correlated genes. Heatmap correlation analysis of DEGs (>2-fold changes with significance level at 0.05) showed significant associations with AF-fed *Bos taurus*. For example, six DEGs from the adipose tissue showed strong positive correlations with AF-fed animals, while five DEGs were negatively correlated with AF-fed animals compared to the NF group, which tended to show a significance at a *p* < 0.001–0.047 level (Figure 1e). Four DEGs from liver tissues were more strongly correlated with AF-fed animals, while three DEGs were negatively correlated with the AF-fed animals at a significance level of *p* < 0.002–0.044 (Figure 1f). In muscle tissues, six DEGs were positively correlated with AF-fed animals, while one DEG was negatively correlated with the AF-fed animal group, which tended to show a significance at *p* < 0.001–0.042 (Figure 1g).

### 2.3. Differentially Expressed Genes (DEGs) and Pathways

Functional annotation of DEGs was performed with a DAVID Bioinformatics tool. Functional analysis of DEGs in adipose tissues of *Bos taurus* revealed that endodermal and skeletal muscle cell differentiation, cellular response to calcium ion, T cell migration, process associated with osteoclast differentiation, chemokine production, and wound healing were modulated by divergence in AF-fed animals (Supplementary Table S1). Functional analysis of DEGs in liver tissues indicated that antigen processing and presentation of endogenous immune response, acute phase response, biosynthetic pathways along with osteoblast differentiation processes, and negative regulation of transcription from RNA polymerase could be regulated by variations in AF-fed animals (Supplementary Table S2). A functional analysis of muscle tissues of *Bos taurus* revealed DEGs closely associated with the processing and presentation of endogenous antigens, immune responses, and positive regulation of transcription by RNA polymerase and cellular responses to starvation toward divergence in AF-fed animals (Supplementary Table S3). All DEGs were successfully mapped to a molecular or biological pathway and/or category in the Kyoto Encyclopedia of Genes and Genomes (KEGG) pathway database. All DEGs were analyzed and separated according to their biological functions with KEGG. KEGG signaling enrichment analysis results of DEGs in adipose, liver, and muscle tissues of *Bos-Taurus* are presented in Supplementary Tables S4–S6. These enriched pathways were related to osteoclast differentiation, phagosome, TNF signaling, PI3K-Akt signaling pathways, non-alcoholic fatty liver diseases, T cell leukemia virus-1 infection, Epstein Barr virus infection, NF-kappa B signaling cascade, MAPK signaling pathway, cellular senescence, viral carcinogenesis, and Kaposi sarcoma-associated herpesvirus infection.

### 2.4. Sequencing Depth, Coverage, and α-Diversity Index of Bacteria and Fungi

A total of 108,701 V3-V4 16S sequence reads were obtained from eight samples after filtering low quality, chimera, and other illegible data (minimum reads of 9494 to maximum reads of 19,232). The average number of operational taxonomic unit (OTUs) detected by the analysis was 459.9 for each experimental sample (NF-458.3 vs. AF-422.3 OTU). For fungi, a total of 762,598 ITS reads from eight samples were recorded with a mean of 95,324 reads for each sample (minimum reads of 48,461 to maximum reads of 113,015). The average number of OTUs detected by the analysis was 83 for each experimental sample (NF-73.6 vs. AF-98.3OTU). The experimental sample had greater good coverage values above 99% for both bacteria and fungi of each sample, indicating that sampling data were sufficiently representative of bacteria in NF- and AF-fed animal groups. Cho1, Shannon, and Gini-Simpson indexes showed significant differences between NF- and AF-fed animals, indicating that the microbial community was more diverse and richer (Supplementary Table S7).

### 2.5. Rumen Microbiota Community Compositions of NF- and AF-Fed Animals

**Figure 2:**
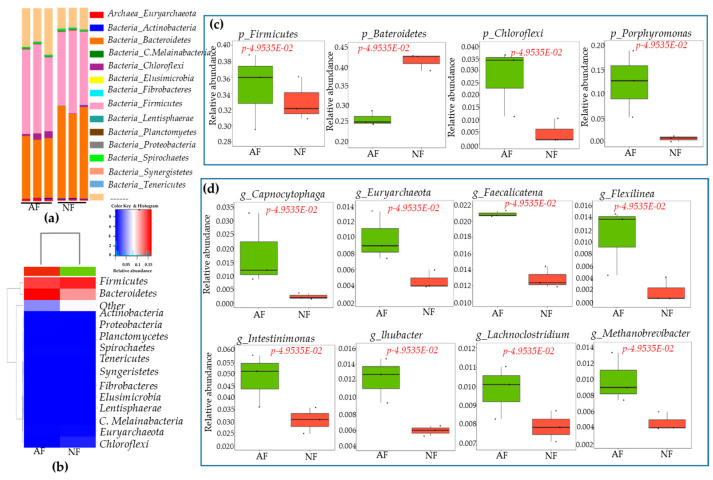
Changing bacterial dynamics in rumen samples of cattle in response to dietary variations. The bar diagram indicates relative abundance of bacteria in the rumen sample at phylum level (**a**). To understand the relationships between bacteria at the phylum level and dietary supplements, Pearson correlation coefficients were generated using an R software-3.6.2 (**b**). The boxplot graphs show the difference in phylum (**c**) or genus level (**d**) in experimental samples at a significant level < 0.05.

At the phylum level of bacteria, a total 15 phyla were identified in rumen samples. *Firmicutes*, *Bacterioidetes*, *Chloroflexi*, and *Porphyromonas* were the dominant phyla among these fifteen phyla (Figure 2a). Heatmap correlation between bacteria at the phylum level and dietary variations showed significant differences in relative abundance between NF- and AF-fed animal groups (Figure 2b). Boxplot graphs showed significant differences in phylum- and genus-level bacterial communities between animals fed NF and AF. Animals fed AF had a higher abundance of *Firmicutes*, *Chloroflexi*, and *Porphyromonas* but lower *bacterioidetes* abundance than NF-group animals (Figure 2c). At the genus level of bacteria, a total 14 genera were identified in rumen samples. Among these identified genera, *Capnocytophaga*, *Euryarchaeota*, *_Faecalicatena*, *Flexilinea*, *Intestinimonas*, *lhubacter*, *Lachnoclostridium* and *Methanobrevibacter* were dominant genera found in experimental samples at significant levels (Figure 2d). Animals fed with AF showed a higher abundance of *Capnocytophaga*, *Euryarchaeota*, *_Faecalicatena*, *Flexilinea*, *Intestinimonas*, *_lhubacter*, *Lachnoclostridium* and *Methanobrevibacter* than NF animals (*p* < 0.05).

**Figure 3:**
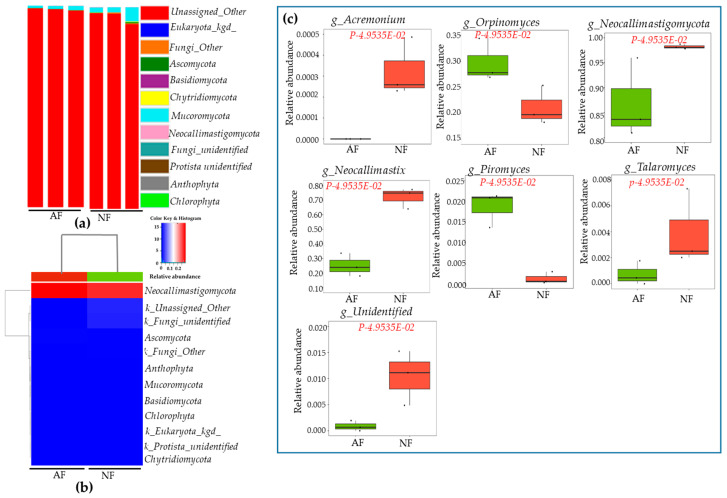
Changing bacterial dynamics in rumen samples of cattle in response to dietary variations. The animal was fed a normal diet feed (NF) and alternative diet feed (AF) until patenting. An analysis of metagenomics was performed on rumen samples of cattle using the pyrosequencing method. The ITS regions were sequenced. The bar diagram indicates relative abundance of fungi in the rumen sample at phylum level (**a**); to understand the relationships between bacteria at the phylum level and dietary supplements, Pearson correlation coefficients were generated using an R software-3.6.2 (**b**). The boxplot graphs show the difference at genus level (**c**) in experimental samples at a significant level < 0.05.

**Figure 4:**
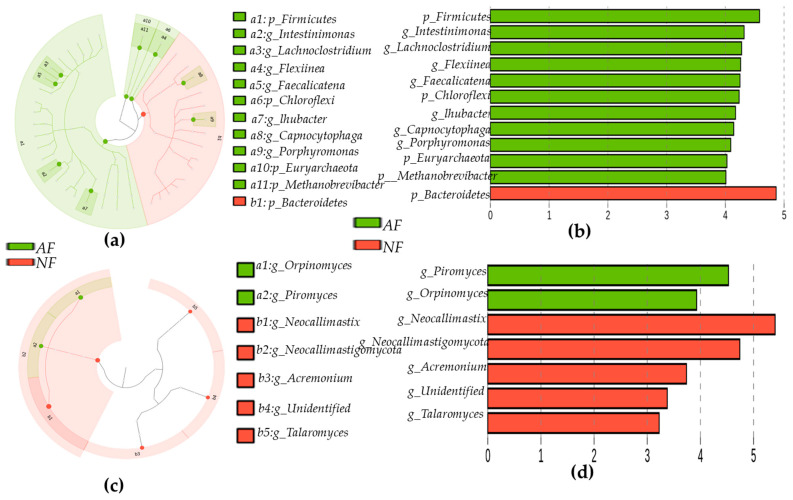
The phylogenetic distribution of bacteria and fungi in rumen samples by diet variations. The phylogenetic distribution of bacteria and fungi associated with rumen samples is represented by a cladogram, lineages with LDA values of default range 2 determined by linear discriminant analysis (LEfSe). Differences are indicated by the color of a class. Red color indicates bacteria at phylum and genus levels in rumen of a normal-feed (NF) diet group of cattle, while green color indicates bacteria at phylum and genus levels in rumen of an alternative-feed (AF) diet group of cattle. The taxonomic levels are abbreviated from phylum to genus as alphabets and numbers (a1–11 and b1), which are labeled before microbial groups. Bacteria distribution at the phylum and genus level in experimental samples (**a**,**b**); fungi distribution at genus level in AF and NF groups (**c**,**d**).

Regarding fungi at the phylum level, a total 12 phyla were identified in rumen samples. There was no significant difference in fungi at the phyla level between NF- and AF- fed animals (Figure 3a). Heatmap correlation between bacteria at the genus level and dietary variations are presented in Figure 3b. However, boxplot graphs showed significant differences in genus-level bacterial communities between animals fed NF and AF (Figure 3c). The genus level *Acremonium*, *Orpinomyces*, *Neocallimastigomycot*, *Neocallimastix*, *Piromyces*, and *Talaromyces* were dominant in rumen samples at significant levels (*p* < 0.05). *Acremonium*, *Neocallimastix*, *Talaromyce*, and other fungi were more abundant in the NF group, whereas *Orpinomyces* and *Piromyces* were more abundant in the AF group (*p* < 0.05). Furthermore, linear discriminant analysis (LEfSe) and LDA were used to determine the microbial taxonomic difference from phylum to genus. Results showed significant differences in bacteria and fungi between NF-and AF-fed animals. These data are shown in cladograms. LDA scores > 2 were confirmed by LEfSe (Figure 4a–d).

### 2.6. Heatmap Correlation Studies Between DEGs and Bacteria or Fungi

**Figure 5:**
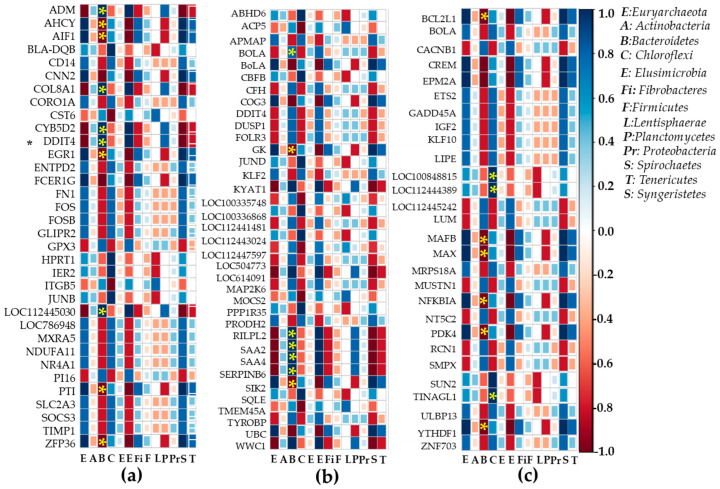
The interaction study between DEGs in major metabolic organs and rumen bacteria. An analysis was conducted using R software 3.6.2 to correlate relative abundances of bacteria at the phylum level in rumen with differentially expressed genes (greater than 1.5-fold changes at the *p* < 0.05 level) in adipose, liver, and muscle tissues. (**a**) Interaction between differentially expressed genes in adipose tissue and rumen bacteria at phylum level; (**b**) interaction between differentially expressed genes in liver tissue and rumen bacteria at phylum level; (**c**) interaction between differentially expressed genes in muscle tissue and rumen bacteria at phylum level. The marked asterisk indicates a significant correlation between DEGs and bacteria or fungi at the *p* < 0.05 level, reflecting either positive or negative interactions.

**Figure 6:**
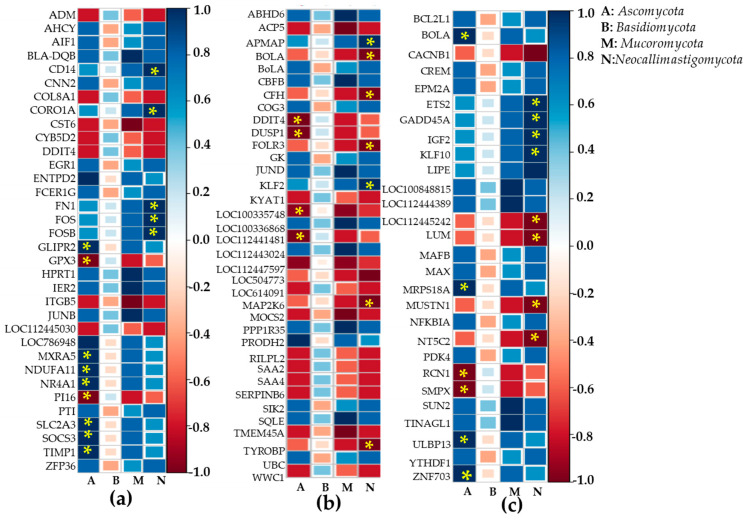
An interaction study between DEGs in major metabolic organs and rumen bacteria by R software-3.6.2 version. The relative abundance of fungi at the phylum level in rumen was correlated with differentially expressed genes (greater than 1.5-fold changes at the 0.05 level) in adipose, liver, and muscle tissues. (**a**) An interaction between differentially expressed genes in adipose tissue and rumen fungi at the phylum level; (**b**) an interaction between differentially expressed genes in liver tissue and rumen fungi at the phylum level; (**c**) an interaction between differentially expressed genes in muscles and rumen fungi at phylum level. The marked asterisk indicates a significant correlation between DEGs and bacteria or fungi at the *p* < 0.05 level, reflecting either positive or negative interactions.

Correlations between rumen bacteria and differentially expressed genes in adipose tissue (Figure 5a), liver tissue (Figure 5b), and muscle tissue (Figure 5c) were analyzed. Firmicutes phyla did not correlate significantly with DEGs. Other major phyla *Bacterioidetes* were positively correlated with DEGs such as *PI16, LOC112445030, DDIT4, CYB5D2, COL8A1*, and *ADM*, but negatively correlated with *ZFP36, PTI, EGR1, ACHY*, and *AID1* (*p* < 0.05). DEGs such as *BoLA, SIK2*, and *GK* from liver tissues were negatively correlated with *Bacterioidetes*, whereas *RILPL2, SAA2,* and *SAA4* were positively correlated. *Bacterioidetes* showed negative correlations with DEGs such as *BCL2L1, CREM, EPM2A, MAFB, MAX*, and *YTHDF1* in muscle tissues (*p* < 0.05). *Chloroflexi* showed significant correlations with *BLA-DQB*, *HPRT1*, *IER2*, *JUNB*, *and CST6 DEGs.* They were negatively associated with *TMEM45A, MOCS2, LOC112447597, LOC100335748*, and *ACP5*, but positively associated with *SQLE* and *CBFC* in liver tissues (*p* < 0.05). DEGs from muscle tissues (*SUN2, TINAGL1, LOC100848815*, and *LOC112444389*) were positively correlated with *chloroflexi.* A heatmap correlation study between DEGs of adipose tissues (Figure 6a), liver tissues (Figure 6b), muscle tissues (Figure 6c), and fungi was performed. DEGs such as *TIMP1, SOCS3, SLC2A3, GLIPR2, MXRA5, NDUFA11*, and *NR4A1* from adipose tissues and *ZNF703, ULBP13, MRPS18A, BOLA* from muscle tissues were positively correlated (*p* < 0.05). *PI16* and *GPX3* from adipose tissues, *DDIT4, DUSP1, LOC100335748*, and *LOC112441481* from liver tissues, *RCN1* and *SMPX* from muscle tissues correlated negatively with *Ascomycota* (*p* < 0.05). DEGs in adipose tissues (*FOS, FOSB, FNI CORO1A CD14*), liver tissues (*KLF2*), and muscle tissues (*LIPE, KLF10, IGF2, GADD45A, ETS2*) were positively correlated with *Neocallimastigomycota* (*p* < 0.05). In contrast, liver DEGs (such as *TYROBP, MAP2K6, FOLR3,* and *BOLA*) and muscle DEGs (such as *NT5C2, MUSTN1,* and *CACNB1*) were negatively correlated with *Neocallimastigomycota* (*p* < 0.05).

## 3. Discussion

The cost of feed accounts for a substantial portion of cattle farming expenses [27]. Thus, increasing feed efficiency, reducing costs, and enhancing profitability will require optimizing feeding strategies (such as feed formulation). To reduce the cost of production, a new strategy is needed to reduce the cost by using alternative feed ingredients, such as crop residues and industrial byproducts. Moreover, such a strategy can reduce the environmental footprint associated with feed production [28]. Fermented feed production with inoculants, such as lactic acid bacteria and yeast, can improve nutrients and reduce anti-nutritional substances [29]. The production and optimization of feed using residual byproducts, such as distillers dried grains, corn gluten feed, and rice bran, with their impact on animal performance, have been previously studied, with results suggesting that animals fed alternate diets (fermented feed) perform significantly better than those fed normal diets [6]. In the current study, fermented grain feed was produced from agricultural crop byproducts that substituted *Italian ryegrass* and whole crop corn silage (Alternative feed: AF). An investigation of the effects of alternative feed on animal performance suggested that AF diets significantly improved animal performance over normal-feed diets [6]. These findings aligned with previous reports suggesting that fermented feeds could improve nutrient utilization and gut health, thereby promoting growth and meat yield in beef cattle [30]. Continuing the study, we further explored the transcriptome changes in different tissues, such as adipose, liver, muscle tissues, and microbial changes, in rumen samples of cattle. The functional enrichment analysis revealed GO biological processes and pathways influenced by diet variations. A specific diet induced changes in expression of genes associated with a specific biological process and pathway. DEGs’ functions in adipose, liver, and muscle tissues are mostly related to transcription regulation by RNA polymerase II promoter, differentiation of endothelial and skeletal muscles, calcium ion response, T cell migration, fibroblast growth factor stimulation, osteoclast differentiation, and chemokine production. A deeper understanding of the mechanisms underlying diet variation can be gained through these pathways. Key regulators that may affect the expression of target genes in response to changes in nutrient availability have been reviewed [31]. Data showed differentially expressed genes between different diet groups. *ADM, COL8A1, CYB5D2, GPX3*, and *ZFP36* were DEGs upregulated more than two-fold in adipose tissues of animals fed with the alternative diet, whereas DEGs such as *BLA-DQB, CXCL3, EGR1, FOS, FOSB, JUNB, LOC112449175, MXRA5, SOCS3, TIMP1*, and *ZFP36* were downregulated. These transcripts are actively involved in several biological functions, such as immune-related pathways [32,33,34] and muscle growth, and play an important role in regulating muscle hypertrophy [35,36,37,38], regulation of adipose tissue, inflammatory response [39,40,41], ovulation, embryogenesis, angiogenesis, wound healing, extracellular matrix remodeling [42], and cellular metabolism [43]. Liver transcriptome analysis revealed that *ABHD6, BOLA, LOC112447597*, and *PPP1R1A* were upregulated while *CCL19, FOLR3, KYAT1, LOC100335748, LOC100336868, LOC112443024*, *TMEM45A*, and *TYROBP* were downregulated in response to alternative diet feeding. These DEGs are involved in inflammation/immune-related functions [44], beta cell regulation [45], and metabolic pathways [46]. Alternative diet supplementation significantly altered a variety of DEGs in muscle tissues, including *ATF3*, *C1QTNF3, LIPE, LOC100848815, LOC104973829, LOC112444389, LOC112445242, LUM, MRPS18A, PDK4, POSTN, PPIF,* and *ULBP13*. These DEGs have been shown to modulate metabolic processes, immunity, and oncogenesis [47], immune functions [48], collagen fibril structure [49], and fatty acid metabolism [50,51].

Animal performance and health are directly affected by rumen microbial communities [52,53]. The goal of the current study was to determine effects of diets on microbial communities, such as bacteria and fungi, in cattle rumen by performing barcode pyrosequencing of hypervariable 16S rRNA regions and investigating the relationship between microbes and different expressions of genes in tissues. A significant difference in alpha diversity index or relative abundance of the main phyla or genera was found between NF and AF groups in this study, indicating that alternative diets could significantly affect microbial dynamics at the phylum and genus levels. Dominant phyla were *Firmicutes*, *Bacterioidetes*, *Chloroflexes*, and *Porphyromonas* (*p* < 0.05) in both *NF* and AF groups. *Firmicutes, Chloroflexi*, and *Porphyromonas* were more abundant in AF-fed animals, whereas the abundance of *Bacterioidetes* was lower in the AF group. The enrichment of *Firmicutes* and *Chloroflexi* in AF-fed animals may indicate enhanced ability for fiber degradation and utilization of specific substrates. A majority of *Firmicutes* are composed of gram-positive bacteria with low G + C contents [54,55]. *Ruminococcus*, *Streptococcus*, *Flintibactor*, *intestinimonas*, *Christensenella*, *Butyrivibrio*, *Faecalicatena*, *Lachnoclostridium*, *Syntrophococcus*, *Sporobacter*, *Succiniclasticum*, *Capnocytophaga*, *Faecalicatena*, and *Methanobrevibacter* are the most abundant genera in *Firmicutes phyla*, accounting for 39–42% (Supplementary Table S8). Alternative diets increased *Faecalicatena*, *Intestinimonas*, *Streptococcus*, and *Lachnoclostridium*. The relative abundance of *Intestinimonas* decreases linearly in response to dietary variations [56]. Despite this, the AF diet supplement resulted in an increase in *Intestinimonas* abundance (Supplementary Table S9). This genus can produce butyrate and acetate from lysine and sugar [57,58]. For *Bacterioidetes*, another major phylum (31.9 to 46.4%), *Lentimicrobium*, *Prevotella*, and *Barnesiella* were more abundant in the AF group, indicating that diet alteration could significantly influence both phylum and genus. Several *Prevotella* species are present in rumen microbial communities. They are predominantly found in animals fed high-grain diets [14,59]. *Prevotella* uses starches, non-cellulosic polysaccharides, and simple sugars as energy sources for succinate production [59]. It possesses hemi-cellulolytic and proteolytic activities [60]. Fungal populations can also be influenced by diet [61]. They can secrete various cell wall-degrading enzymes such as free enzymes and multi-enzyme complexes [62], which are capable of degrading plant biomass effectively [63]. The present study determined rumen fungi population in response to an alternative diet. *Neocallimastigomycota* accounted for 98.2% and 86.88% in NF and AF groups, respectively (Supplementary Table S10). *Ascomycota* was the second most dominant phyla in both NF and AF groups. *Neocallimastigomycota* was mainly composed of *Orpinomyces*, *Piromyces*, and other species. AF increased *Orpinomyces* (from 21.15% to 29.7%), *Piromyces* (from 0.1% to 1.8%%), and other fungi (from 4.2% to 24.9%), but reduced *Neocallimastix* (from 72.0% to 25.2%) abundances (Supplementary Table S11). These fungi (*Orpinomyces* and *Neocallimastix*) are actively involved in cell wall digestion more efficiently [64]. Similarly, animals fed grains had a higher abundance of *Orpinomyces* than those fed fiber diets [65]. Several enzyme mixtures can be produced by *Piromyces* through its metabolic pathway and used to digest plant cell wall components’ degradation [66]. LDA and LEfSe analyses supported the current finding, confirming that key microbial taxa differentiated between NF and AF diet treatments. The microbial shifts observed might have most important implications for feed efficiency, and fermentation end-products.

Finally, this study determined whether there was a correlation between the relative abundance of rumen bacteria and differentially expressed genes in adipose, liver, and muscle tissues. A correlation analysis was conducted. Correlation revealed that *Firmicutes* phyla did not correlate significantly with DEGs in different tissues. In adipose tissues, other major phyla *Bacterioidetes* were positively correlated with DEGs such as *PI16*, *LOC112445030, DDIt4, CYB5D2, COL8A1,* and *ADM* but negatively correlated with ZFP36, *PTI, EGR1, ACHY*, and *AID1*. In the liver, DEGs such as *BoLA, SIK2*, and *GK* were negatively correlated with *Bacterioidetes* while *RILPL2, SAA2*, and *SAA4* were positively correlated with *Bacterioidetes*. In the muscle, *Bacterioidetes* showed negative correlations with DEGs such as *BCL2L1, CREM, EPM2A, MAFB, MAX*, and *YTHDF1*. *BLA-DQB, HPRT1, IER2, JUNB, and CST6 DEG* were positively correlated with Chloroflexi in adipose tissues. They were negatively associated with *TMEM45A, MOCS2, LOC112447597, LOC100335748,* and *ACP5*, but positively associated with *SQLE* and *CBFC* in liver tissues. *Chloroflexi* were positively correlated with DEGs (*SUN2, TINAGL1, LOC100848815*, and *LOC112444389)* from muscle tissues, suggesting that microbial changes in the rumen could alter gene expression patterns in different tissues. Moreover, DEGs from different tissues were correlated with phyla of fungi such as *Ascomycota*, *Basidiomycota*, *Mucoromycota*, and *Neocallimastigomycota*. Data revealed that *TIMP1, SOCS3, SLC2A3, GLIPR2, MXRA5, NDUFA11*, and *NR4A1* from adipose tissues and *ZNF703, ULBP13, MRPS18A*, and *BOLA* from muscle tissues were positively correlated with *Ascomycota*. However, PI16 and GPX3 from adipose tissues, *DDIT4, DUSP1, LOC100335748*, and *LOC112441481* from liver tissues, and *RCN1* and *SMPX* from muscle tissues were negatively correlated with *Ascomycota.* DEGs in adipose tissue (*FOS, FOSB, FNI CORO1A CD14*), liver tissue (*KLF2*), and muscle tissue (*LIPE, KLF10, IGF2, GADD45A, ETS2*) were positively correlated with *Neocallimastigomycota*. However, liver DEGs (such as *TYROBP, MAP2K6, FOLR3*, and *BOLA*) and muscle DEGs (such as *NT5C2, MUSTN1*, and *CACNB1*) were negatively correlated with *Neocallimastigomycota*. These findings highlight that the microbial composition of rumen can exert its systemic effects on host gene expression across key metabolic organs. The significant correlation between rumen bacteria or fungi and DEGs suggests that microbial metabolites may influence rumen activity through extra-ruminal physiology. Understanding these microbiome–host interactions offers potential strategies to modulate rumen microbiota through diet management to enhance nutrient utilization and improve productivity in animals. Based on the present finding, it is possible that a fermented feed diet derived from agricultural byproducts modulates the microbiome composition in *Bos-taurus* rumen; consequently, it affects host gene expression across metabolic organs such as adipose, muscle, and liver tissues, which leads to improving the utilization of nutrients and animal performance in Hanwoo steers. This hypothesis is supported by observed changes in microbial diversity, particularly *Firmicutes*, *Bacteroidetes*, *Chloroflexi*, and various fungi. These modulations are also closely correlated with differences in gene activity, which are related to immune responses and tissue development. These systemic gene expression changes may be mediated by microbial metabolites, suggesting that dietary modulation of the microbiome could enhance livestock productivity and reduce feed costs. However, further research is required to explore longer-term effects of fermented feed on Hanwoo steers across a larger cohort and diverse production settings. The focus of future work should be on validating the functional relevance of key differentially expressed genes through targeted qPCR or protein-level analysis to gain deeper mechanistic insights.

## 4. Materials and Methods

### 4.1. Crops Collection and Production of Silage with Bacteria

Crops such as Italian ryegrass (IRG) and whole crop corn (WCC) were cultivated in Jeollabuk province, Republic of Korea. Early flowering IRG and yellow ripened WCC were harvested and wilted for two days in the field. Crop moisture levels were monitored frequently. *L. plantarum-46*, *L. plantarum-KCC-10*, and *L. plantarum-KCC-19* were then added with an automatic spraying machine after reaching 60 to 65% moisture and packed in round bales. A mixture of *L. plantarum* in sterile distilled water was prepared as described previously [67].

### 4.2. Fermented Feed Preparation

Fermented feed (Alternate diet feed: AF) was prepared by Inbio Corporation Korea based on National Institute of Animal Science feed composition standards. Distillers dried grains, rice bran, cornflakes, corn gluten, limestone, and vitamin premix were combined with different microbial strains, such as *B. subtilis*, *S. cerevisiae*, and *L. plantarum*, and incubated at 25 °C for six days under aerobic conditions to initiate fermentation. AF was then transferred to a feed mixer with a conveyor system (DW: 300-22, Dongwoo solution corporation, Busan, Republic of Korea).

### 4.3. Ethics Statements

The study was conducted in accordance with relevant guidelines and regulations of the National Institute of Animal Science, Rural Development Administration, Republic of Korea (Ethical approval number: NIAS 2020-443/17 February 2020). All experimental protocols adhered to the Animal Research: Reporting in Vivo Experiments (ARRIVE) guidelines.

### 4.4. Animal and Experimental Design

A group of 13-month-old Hanwoo steers (Genus: *Bos*; Species: *Bos-taurus*; Color: brown coat) was obtained from Daum Hanwoo farm in Jeollabuk province located nearby the National Institute of Animal Science, Jeonju, Republic of Korea. These animals were divided into two groups: a normal-feed (NF) diet group and an alternative-feed (AF) diet group. There were six animals (n = 6) in each group. Average initial body weights were 376.4 kg (ranging from 232 kg to 420 kg) for normal-feed diet animals and 415 kg (ranging from 335 kg to 449 kg) for alternate-feed diet animals. According to Korean Feeding Standards, the NF group of Hanwoo steers received rice straw and concentrate, while the AF group of Hanwoo steers received IRG and WCC silage along with fermented grains, for 13 months. As part of early patterning periods, the NF group received rice straw 3.5 kg and concentrate 8 kg, whereas the AF group received IRG silage, WCC silage, and fermented grains (3.0, 5.86, and 7.43 kg, respectively) without concentrate. In late patterning, the NF group of Hanwoo steers received 1.79 kg of rice straw and 12.57 kg of concentrate, while the AF group of Hanwoo steers received 0.79 kg of rice straw, 1.29 kg of IRG silage, 0.43 kg of WCC silage, and 8.86 kg of fermented grains. Ad libitum access to water was provided to all animals. The experimental feed was fed twice daily at 9:00 a.m. and 16:00 p.m. to animals. Average body weight, daily gain, and feed intake were measured [6]. After experimental periods, all animals were slaughtered at the slaughterhouse, Dodram Pvt Ltd., Gyeonggi-do, Republic of Korea (https://auction.dodram.co.kr/facility.html). Adipose, liver, and muscle tissues were then collected from experimental animals. These tissues were immediately placed in liquid nitrogen and transported to the laboratory and kept at −80 °C until use.

### 4.5. RNA Extraction from Different Tissues

Total RNAs were extracted from liver, adipose, and muscle tissues using a Trizol and RNA lipid tissue mini kit (Qiagen, Valencia, CA, USA). The quality of RNA was analyzed with an Agilent 2100 bio-analyzer (Agilent Tech, Waldbronn, Germany). RNA was quantified with an ND-2000 spectrophotometer (Thermo Fisher Scientific, Waltham, MA, USA).

### 4.6. Preparation of Library and Sequencing

RNA libraries were constructed with an NE Next Ultra-II Directional RNA-Sequencing kit (New England Biolabs Inc., Ipswich, MA, USA). A Poly-A RNA selection kit (Lexogen Inc., Vienna, Austria) was used to extract mRNA. cDNA was then synthesized and sheared according to manufacture protocols. Indexing was performed using Illumina indexes 1–12 and enriched by PCR. Libraries were screened with a TapeStation HS D1000 screen Tape (Agilent Tech, Waldbronn, Germany) to determine the fragment size. They were quantified with a stepOne RT-PCR (Life Tech. Inc., Wheeling, IL USA). High-throughput sequencing was performed with a NovaSeq 6000 (Illumina, Inc., San Diego, CA, USA).

### 4.7. Analysis of Data and Removal of Low-Quality Reads

Quality control of raw sequences was carried out using FastQC version 0.12.1 [68]. Adapter reads and low-quality reads were removed with a FASTX_Trimmer (FASTX-Toolkit at cshl.edu) and BBMap [69]. Quality reads were mapped to a reference genome using TopHat [70]. Read count data were processed using the EdgeR’s FPKM+Geometric normalization method in R [71]. Fragments per kb per million reads (FPKM) were calculated using Cufflinks [72]. Data mining and graphic visualization were performed with ExDEGA-5.0.0 (Ebiogen Inc., Seongdong-gu, Seoul, Republic of Korea).

### 4.8. Heatmap Correlation and Gene Ontology

The heatmap correlation between DEGs and diet supplements was generated using the ExDEGA graphicPlus software-5.0.0 version. We generated the heatmap based on changes in genes in adipose, liver, and muscle tissues greater than 2-fold at *p* < 0.05. Functional annotation (biological process, cellular components, and molecular functions) and gene ontology of DEGs were identified using the online DAVID tool (https://davidbioinformatics.nih.gov/summary.jsp (accessed on 24 July 2024)).

### 4.9. Genomic DNA Extraction

Rumen juice samples were collected from experimental animals at the slaughterhouse. Rumen samples were immediately placed in liquid nitrogen and transported to the laboratory and kept at −80 °C until use. Samples were thawed and centrifuged at 9000× *g* for 10 min at 4 °C. DNA was extracted from residues using a DNeasyPowerSoil Kit (Quiagen, Hilden, Germany) according to the manufacturer’s protocol. DNA was quantified using a Quant-IT PicoGree kit (Invitrogen, Carlsbad, MA, USA).

### 4.10. Library Construction and Sequencing

V3 and V4 region libraries were prepared with the Illumina 16S metagenomics library prep kit following the protocol recommended by the manufacturer. In the first step, two nomograms of total DNA were amplified by PCR with reaction buffer, dNTP (1 mM), universal forward and reverse primers (500 nM), and DNA polymerase (Agilent Tech, Santa Clara, CA, USA). The PCR cycle was performed with the following conditions: heat activation 3 min at 95 °C, 25 cycles of 30s at 95 °C, 30s at 55 °C and 30s at 72 °C, and final extension at 72 °C for 5 min. Primers used for the first PCR were as follows:

V3-F, 5′-TCGTCGGCAGCGTCAGATGTGTATAAGAGACAGCCTACGGGNGGCWGCAG-3;
V4-R, 5′ TCTCGTGGGCTCGGAGATGTGTATAAGAGACAGGACTACHVGGGTATCTAATCC-3.
For fungi ITS3 amplicon, PCR forward primer-5
TCGTCGGCAGCGTCAGATGTGTATAAGAGACAGGCATCGATGAAGAACGCAGC-3 and ITS4 amplicon PCR reverse primer-5 GTCTCGTGGGCTCGGAGATGTGTATAAGAGACAGTCCTCCGCTTATTGATATGC-3 were used.

First PCR products were purified using AMPure beads (Agencourt Bioscience, Beverly, MA, USA) and amplified further with PCR for library construction using NexteraXT indexed primers. Conditions for the second PCR were similar to those for the first PCR except that the number of cycles was different. Here, a total of 10 cycles was used. Final products were purified with AMPure beads (KAPA library quantification kit for illumine sequencing platforms). Purified products were quantified by real-time PCR according to the manufacturer’s protocol (Agilent Tech, Waldbronn, Germany). The quality of products was analyzed with a Tapestation D1000 ScreenTape system (Agilent Tech, Waldbronn, Germany). Paired-end (2 × 300 bp) sequences were generated using a MiSeqTM platform (Illumina, San Diego, CA, USA). Poor quality sequences were removed with CD-HIT-OUT/rDNATools.

### 4.11. Determination of Relative Abundance of Microbial Dynamics

To determine relative abundances of microbes in experimental groups, alpha and beta diversity indices were calculated from the complete operational taxonomic unit (OTU) table (alpha_diversity.py; UCLSUT/RDP(16s) or UNITE (ITS); alpha_rarefaction.py; make_2d_plots.py and make_otu_heatmap_html.py). A Pearson correlation coefficient was generated using R software 3.6.2 to understand relationships between microbial dynamics and dietary supplements (Macrogen, Seoul, Republic of Korea). A linear discriminant analysis effect size (LEfSe) was used to distinguish significant differences among communities or phyla.

### 4.12. Integrated Analysis of Interactions Between DEGs in Major Metabolic Organs and Relative Abundance of Bacteria or Fungi in Rumen

We used R software 3.6.2 to correlate relative abundances of bacteria and fungi at the phylum level in rumen with differentially expressed genes (greater than 1.5-fold changes at 0.05 level) in adipose, liver, and muscle tissues [73].

## 5. Conclusions

This study demonstrated that feeding *Bos taurus* with a fermented diet made with *Lactobacillus* sp., *Bacillus* sp., and yeast (AF) significantly enhanced growth performance, final body weight, and modulated gene expression in key metabolic tissues compared to a normal diet (NF). The AF diet also resulted in notable shifts in rumen microbiota, modulating both bacterial and fungal diversity and altering the abundance of specific microbial taxa. These microbial changes were significantly correlated with differentially expressed genes involved in metabolic, immune, and cellular regulatory pathways, suggesting a strong diet–microbiome–host interaction. Based on these findings, it is recommended to further explore the use of fermented diets as a sustainable strategy to improve cattle productivity. Future studies should validate functional roles of identified genes, investigate underlying mechanisms of microbiota–host interactions, and expand the research across different environments and cattle breeds to support precision feeding and health management in livestock.

## Figures and Tables

**Table 1:** Differentially expressed genes (DEGs) in adipose tissue of experimental animals.

S.Nos	Gene_Symbol	Gene Name	Fold	*p* Values
1.	*ADM*	*Adrenomedullin*	0.396	0.017
2.	*AHCY*	*Adenosylhomocysteinase*	1.780	0.010
3.	*AIF1*	*Allograft inflammatory factor 1*	1.806	0.040
4.	*BLA-DQB*	*MHC class II antigen*	4.589	0.014
5.	*CD14*	*CD14 molecule*	1.576	0.046
6.	*CNN2*	*Calponin 2*	1.548	0.034
7.	*COL8A1*	*Collagen type VIII alpha 1 chain*	0.381	0.007
8.	*CORO1A*	*Coronin 1A*	1.648	0.047
9.	*CST6*	*Cystatin E/M*	0.604	0.024
10.	*CYB5D2*	*Cytochrome b5 domain containing 2*	0.494	0.040
11.	*DDIT4*	*DNA damage inducible transcript 4*	0.616	0.036
12.	*EGR1*	*Early growth response 1*	3.915	0.039
13.	*ENTPD2*	*Ectonucleoside triphosphate diphosphohydrolase 2*	1.560	0.036
14.	*FCER1G*	*Fc epsilon receptor Ig*	1.734	0.045
15.	*FN1*	*Fibronectin 1*	1.617	0.007
16.	*FOS*	*Fos proto-oncogene*, *AP-1 transcription factor subunit*	4.819	0.013
17.	*FOSB*	*Fosb proto-oncogene*, *AP-1 transcription factor*	11.001	0.007
18.	*GLIPR2*	*GLI pathogenesis related 2*	1.702	0.049
19.	*GPX3*	*Glutathione peroxidase 3*	0.339	0.028
20.	*HPRT1*	*Hypoxanthinephosphoribosyltransferase 1*	1.611	0.012
21.	*IER2*	*Immediate early response 2*	1.881	0.009
22.	*ITGB5*	*Integrin subunit beta 5*	0.641	0.000
23.	*JUNB*	*Junb proto-oncogene*, *AP-1 transcription factor*	2.319	0.017
24.	*LOC112445030*	*Four and a half LIM domains protein 1-*	0.610	0.037
25.	*LOC786948*	*Tryptase-2-like*	1.811	0.008
26.	*MXRA5*	*Matrix remodeling associated 5*	3.365	0.014
27.	*NDUFA11*	*NADH:ubiquinone oxidoreductase*	1.609	0.020
28.	*NR4A1*	*Nuclear receptor subfamily 4 group A member 1*	3.575	0.010
29.	*PI16*	*Peptidase inhibitor 16*	0.268	0.047
30.	*PTI*	*Pancreatic trypsin inhibitor*	1.974	0.007
31.	*SLC2A3*	*Solute carrier family 2 member 3*	1.696	0.040
32.	*SOCS3*	*Suppressor of cytokine signaling 3*	2.595	0.037
33.	*TIMP1*	*TIMP metallopeptidase inhibitor 1*	2.499	0.014
34.	*ZFP36*	*ZFP36 ring finger protein*	2.106	0.001

**Table 2:** Differentially expressed genes (DEGs) in liver tissue of experimental animals.

S. Nos	Gene_Symbol	Gene Name	Fold	*p* Values
1.	*CL2L1*	*Bcl2 like 1*	1.580	0.033
2.	*BOLA*	*Major histocompatibility complex*, *class i*, *A*	1.996	0.012
3.	*CACNB1*	*Calcium voltage-gated channel auxiliary subunit beta 1*	0.603	0.025
4.	*CREM*	*Camp responsive element modulator*	1.697	0.043
5.	*EPM2A*	*Epm2a glucan phosphatase*, *laforin*	1.535	0.024
6.	*ETS2*	*Ets proto-oncogene 2*, *transcription factor*	1.950	0.030
7.	*GADD45A*	*Growth arrest and DNA damage inducible alpha*	1.804	0.028
8.	*IGF2*	*Insulin like growth factor 2*	1.653	0.035
9.	*KLF10*	*Klf transcription factor 10*	1.536	0.019
10.	*LIPE*	*Lipase e*, *hormone sensitive type*	2.198	0.007
11.	*LOC100848815*	*Sla class ii histocompatibility antigen- d alpha chain-like*	2.872	0.042
12.	*LOC112444389*	*Small nucleolar RNA SNORA32*	27.484	0.013
13.	*LOC112445242*	*Small nucleolar RNA SNORA70*	0.024	0.034
14.	*LUM*	*Lumican*	0.480	0.031
15.	*MAFB*	*Maf bzip transcription factor B*	1.711	0.036
16.	*MAX*	*Myc associated factor x*	1.923	0.039
17.	*MRPS18A*	*Mitochondrial ribosomal protein s18a*	2.114	0.030
18.	*MUSTN1*	*Musculoskeletal*, *embryonic nuclear protein 1*	0.580	0.048
19.	*NFKBIA*	*Nfkb inhibitor alpha*	1.505	0.032
20.	*NT5C2*	*5′-nucleotidase*, *cytosolic ii*	0.638	0.042
21.	*PDK4*	*Pyruvate dehydrogenase kinase 4*	2.733	0.005
22.	*RCN1*	*Reticulocalbin 1*	0.519	0.009
23.	*SMPX*	*Small muscle protein x-linked*	0.663	0.043
24.	*SUN2*	*Sad1 and unc84 domain containing 2*	1.576	0.040
25.	*TINAGL1*	*Tubulointerstitial nephritis antigen like 1*	1.607	0.001
26.	*ULBP13*	*Ul16 binding protein 13*	19.040	0.001
27.	*YTHDF1*	*Yth n6-methyladenosine RNA binding protein f1*	1.597	0.035
28.	*ZNF703*	*Zinc finger protein 703*	1.649	0.008

**Table 3:** Differentially expressed genes (DEGs) in Muscle tissue of experimental animals.

S.Nos	Gene_Symbol	Gene Name	Fold	*p* Values
1.	*ABHD6*	*Abhydrolase domain containing 6*, *acylglycerol lipase*	2.322	0.035
2.	*ACP5*	*Acid phosphatase 5*, *tartrate resistant*	0.623	0.022
3.	*APMAP*	*Adipocyte plasma membrane associated protein*	1.776	0.012
4.	*BoLA*	*Major histocompatibility complex*, *class I*, *antigen*	2.392	0.003
5.	*BoLA*	*Major histocompatibility complex*, *class I*, *A*	0.585	0.025
6.	*CBFB*	*Core-binding factor subunit beta*	1.860	0.036
7.	*CFH*	*Complement factor H*	0.642	0.036
8.	*COG3*	*Component of oligomeric golgi complex 3*	1.828	0.010
9.	*DDIT4*	*DNA damage inducible transcript 4*	0.526	0.009
10.	*DUSP1*	*Dual specificity phosphatase 1*	0.662	0.016
11.	*FOLR3*	*Folate receptor 3*	0.365	0.027
12.	*GK*	*Glycerol kinase*	1.636	0.006
13.	*JUND*	*Jund proto-oncogene*, *AP-1 transcription factor*	1.929	0.042
14.	*KLF2*	*KLF transcription factor 2*	1.898	0.029
15.	*KYAT1*	*Kynurenine aminotransferase 1*	0.303	0.045
16.	*LOC100335748*	*Uncharacterized LOC100335748*	0.024	0.036
17.	*LOC100336868*	*Complement factor H*	3.347	0.035
18.	*LOC112441481*	*Glycine N-phenylacetyltransferase-like*	0.505	0.037
19.	*LOC112443024*	*Uncharacterized*	2.021	0.033
20.	*LOC112447597*	*U6 spliceosomal RNA*	0.016	0.000
21.	*LOC504773*	*Regakine 1*	0.578	0.038
22.	*LOC614091*	*Class I histocompatibility antigen*, *alpha chain BL3*	0.619	0.016
23.	*MAP2K6*	*Mitogen-activated protein kinase kinase 6*	0.621	0.009
24.	*MOCS2*	*Molybdenum cofactor synthesis 2*	0.636	0.007
25.	*PPP1R35*	*Protein phosphatase 1 regulatory subunit 35*	1.596	0.003
26.	*PRODH2*	*Proline dehydrogenase 2*	1.924	0.017
27.	*RILPL2*	*Rab interacting lysosomal protein like 2*	0.629	0.023
28.	*SAA2*	*Serum amyloid A2*	0.597	0.038
29.	*SAA4*	*Serum amyloid A4*, *constitutive*	0.550	0.003
30.	*SERPINB6*	*Serpin peptidase inhibitor*, *clade B* (*ovalbumin*), *member 6*	0.620	0.047
31.	*SIK2*	*Salt inducible kinase 2*	1.747	0.027
32.	*SQLE*	*Squalene epoxidase*	1.598	0.034
33.	*TMEM45A*	*Transmembrane protein 45A*		0.002
34.	*TYROBP*	*Transmembrane immune signaling adaptor TYROBP*	0.418	0.011
35.	*UBC*	*Ubiquitin C*	1.678	0.015
36.	*WWC1*	*WW and C2 domain containing 1*	0.652	0.016

## Data Availability

Sequences of mRNA from adipose, muscle, and liver tissues were submitted to NCBI in the Gene Expression Omnibus (GEO). GEO accession number for mRNA from all tissues was GSE277211 (https://www.ncbi.nlm.nih.gov/geo/query/acc.cgi?acc=GSE277211 accessed on 19 August 2024). All other experimental data are available in the main manuscript and the Supplementary Materials.

## Supplementary Materials

The following supplementary files can be downloaded at: https://www.mdpi.com/article/10.3390/ijms26146816/s1.

## Abbreviations

*ABHD6*	*Abhydrolase domain containing 6: acylglycerol lipase*
*ACP5*	*Acid phosphatase 5, tartrate resistant*
*ADM*	*Adrenomedullin*
*AF*	*Alternative diet feed*
*AHCY*	*Adenosylhomocysteinase*
*AIF1*	*Allograft inflammatory factor 1*
*APMAP*	*Adipocyte plasma membrane associated protein*
*BLA-DQB*	*MHC class II antigen*
*BOLA*	*Major histocompatibility complex, class i,* *A*
*CACNB1*	*Calcium voltage-gated channel auxiliary subunit beta 1*
*CBFB*	*Core-binding factor subunit beta*
*CD14*	*CD14 molecule*
*CFH*	*Complement factor H*
*CL2L1*	*Bcl2 like 1*
*CNN2*	*Calponin 2*
*COG3*	*Component of oligomeric golgi complex 3*
*COL8A1*	*Collagen type VIII alpha 1 chain*
*CORO1A*	*Coronin 1A*
*CREM*	*Camp responsive element modulator*
*CST6*	*Cystatin E/M*
*CYB5D2*	*Cytochrome b5 domain containing 2*
*DDIT4*	*DNA damage inducible transcript 4*
*DEGs*	*Differentially expressed genes*
*DM*	*Dry matter content*
*DUSP1*	*Dual specificity phosphatase 1*
*EGR1*	*Early growth response 1*
*ENTPD2*	*Ectonucleoside triphosphate diphosphohydrolase 2*
*EPM2A*	*Epm2a glucan phosphatase, laforin*
*ETS2*	*Ets proto-oncogene 2, transcription factor*
*FCER1G*	*Fc epsilon receptor Ig*
*FCR*	*Feed conversion ratios*
*FN1*	*Fibronectin 1*
*FOLR3*	*Folate receptor 3*
*FOS*	*Fos proto-oncogene, AP-1 transcription factor subunit*
*FOSB*	*Fosb proto-oncogene, AP-1 transcription factor*
*GADD45A*	*Growth arrest and DNA damage inducible alpha*
*GK*	*Glycerol kinase*
*GLIPR2*	*GLI pathogenesis related 2*
*GO*	*Gene Ontology*
*GPX3*	*Glutathione peroxidase 3*
*HPRT1*	*Hypoxanthinephosphoribosyltransferase 1*
*IER2*	*Immediate early response 2*
*IGF2*	*Insulin like growth factor 2*
*ITGB5*	*Integrin subunit beta 5*
*JUNB*	*Junb proto-oncogene, AP-1 transcription factor*
*JUND*	*Jund proto-oncogene, AP-1 transcription factor*
*KEGG*	*Kyoto Encyclopedia of genes and Genomes*
*KLF10*	*Klf transcription factor 10*
*KLF2*	*KLF transcription factor 2*
*KYAT1*	*Kynurenine aminotransferase 1*
*LEfSe*	*Linear discriminant analysis Effect Size*
*LIPE*	*Lipase e, hormone sensitive type*
*LOC100335748*	*Uncharacterized LOC100335748*
*LOC100336868*	*Complement factor H*
*LOC100848815*	*Sla class ii histocompatibility antigen- d alpha chain-like*
*LOC112441481*	*Glycine N-phenylacetyltransferase-like*
*LOC112443024*	*Uncharacterized*
*LOC112444389*	*Small nucleolar RNA SNORA32*
*LOC112445030*	*Four and a half LIM domains protein 1-*
*LOC112445242*	*Small nucleolar RNA SNORA70*
*LOC112447597*	*U6 spliceosomal RNA*
*LOC504773*	*Regakine 1*
*LOC614091*	*Class I histocompatibility antigen, alpha chain BL3*
*LOC786948*	*Tryptase-2-like*
*LUM*	*Lumican*
*MAFB*	*Maf bzip transcription factor B*
*MAP2K6*	*Mitogen-activated protein kinase kinase 6*
*MAX*	*Myc associated factor x*
*MOCS2*	*Molybdenum cofactor synthesis 2*
*MRPS18A*	*Mitochondrial ribosomal protein s18a*
*MUSTN1*	*Musculoskeletal, embryonic nuclear protein 1*
*MXRA5*	*Matrix remodeling associated 5*
*NDUFA11*	*NADH:ubiquinone oxidoreductase*
*NF*	*Normal diet feed*
*NFKBIA*	*Nfkb inhibitor alpha*
*NR4A1*	*Nuclear receptor subfamily 4 group A member 1*
*NT5C2*	*Nucleotidase 5′, cytosolic ii*
*OUT*	*Operational taxonomic unit*
*PDK4*	*Pyruvate dehydrogenase kinase 4*
*PI16*	*Peptidase inhibitor 16*
*PPP1R35*	*Protein phosphatase 1 regulatory subunit 35*
*PRODH2*	*Proline dehydrogenase 2*
*PTI*	*Pancreatic trypsin inhibitor*
*PUFA*	*Polyunsaturated fatty acids*
*RCN1*	*Reticulocalbin 1*
*RILPL2*	*Rab interacting lysosomal protein like 2*
*SAA2*	*Serum amyloid A2*
*SAA4*	*Serum amyloid A4, constitutive*
*SERPINB6*	*Serpin peptidase inhibitor, clade B (ovalbumin), member 6*
*SIK2*	*Salt inducible kinase 2*
*SLC2A3*	*Solute carrier family 2 member 3*
*SMPX*	*Small muscle protein x-linked*
*SOCS3*	*Suppressor of cytokine signaling 3*
*SQLE*	*Squalene epoxidase*
*SUN2*	*Sad1 and unc84 domain containing 2*
*TIMP1*	*TIMP metallopeptidase inhibitor 1*
*TINAGL1*	*Tubulointerstitial nephritis antigen like 1*
*TMEM45A*	*Transmembrane protein 45A*
*TMR*	*Total mixed ration*
*TYROBP*	*Transmembrane immune signaling adaptor TYROBP*
*UBC*	*Ubiquitin C*
*ULBP13*	*Ul16 binding protein 13*
*VFA*	*Volatile fatty acids*
*WWC1*	*WW and C2 domain containing 1*
*YTHDF1*	*Yth n6-methyladenosine RNA binding protein f1*
*ZFP36*	*ZFP36 ring finger protein*
*ZNF703*	*Zinc finger protein 703*
