# Supplementary material for: Dietary Fermentation with Lactobacillus sp. and Bacillus sp. Modulates Rumen Transcriptomic and Microbiota Profiles in Bos taurus
Source: Int J Mol Sci. 2025 Jul 16;26(14):6816. doi: 10.3390/ijms26146816 (PMC12294822; doi:10.3390/ijms26146816)
Supplement: Supplementary file 1 [file ijms-26-06816-s001.zip › ijms-3688760-supplementary.pdf]

Dietary Fermentation with *Lactobacillus* sp. and *Bacillus* sp. Modulates Rumen Transcriptomic and Microbiota Profiles in *Bos taurus*

Jeong Sung Jung [1,†], Dahye Kim [2,†], Anand Singaravelu [3], Ilavenil Soundharrajan [1,*] and Ki Choon Choi [1,*]

1 Forage Production System Division, National Institute of Animal Science, RDA, Cheonan 31000, Republic of Korea; jjs3873@korea.kr

2 Animal Genomics and Bioinformatics Division, National Institute of Animal Science, Rural Development Administration (RDA), Wanju 55365, Republic of Korea; dhkim0724@korea.kr

3 Department of Chemistry, Saveetha Engineering College, Thandalam 602105, Tamil Nadu, India; anands@saveetha.ac.in

* Correspondence: ilavenil@korea.kr (I.S.); choiwh@korea.kr (K.C.C.); Tel.: +82-41-580-6756 (I.S.); +82-41-580-6752 (K.C.C.); Fax: +82-41-580-6779 (K.C.C.)

† These authors contributed equally to this work.

Supplementary Table S1. Functional annotation of differentially expressed transcripts in adipose tissue between control and FF fed *Bos taurus*

Term	Count	Percent	P-Value	Fold Enrichment
Endodermal cell differentiation	3	8.8	8.00E-04	69.00
Skeletal muscle cell differentiation	3	8.8	2.20E-03	41.70
Cellular response to calcium ion	3	8.8	7.70E-03	22.00
Positive regulation of T cell migration	2	5.9	2.20E-02	88.10
Cellular response to fibroblast growth factor stimulus	2	5.9	2.90E-02	66.10
Positive regulation of transcription from RNA polymerase II promoter	5	14.7	3.70E-02	3.80
Positive regulation of cell differentiation	2	5.9	3.80E-02	50.40
Osteoclast differentiation	2	5.9	5.00E-02	37.80
Positive regulation of chemokine production	2	5.9	5.00E-02	37.80
Antigen processing and presentation of exogenous peptide antigen via MHC class II	2	5.9	5.50E-02	34.10
Positive regulation of primirna transcription from RNA polymerase II promoter	2	5.9	6.60E-02	28.60
Positive regulation of T cell proliferation	2	5.9	7.40E-02	25.20
Wound healing	2	5.9	8.80E-02	21.20

Supplementary Table S2. Functional annotation of differentially expressed transcripts in liver tissue between control and FF fed *Bos Taurus.*

Term	Count	Percent	P-Value	Fold Enrichment
Antigen processing and presentation Of endogenous peptide antigen	8	19	9.60E-16	2.35E+02
Antigen processing and presentation	8	19	1.80E-12	9.17E+01
Immune response	9	21.4	4.40E-08	1.63E+01
Acute-phase response	2	4.8	3.70E-02	52.2
Biosynthetic process	2	4.8	3.90E-02	49.5
Osteoblast differentiation	2	4.8	1.20E-01	14.9
Negative regulation of transcription From rna polymerase ii promoter	4	9.5	1.20E-01	3.2

.

Supplementary Table S3. Functional annotation of differentially expressed transcripts in muscle tissue between control and FF fed *Bos Taurus.*

Term	Count	Percent	P-Value	Fold Enrichment
antigen processing and presentation of endogenous peptide antigen via MHC class Ib	8	22.9	2.20E-16	282
antigen processing and presentation	8	22.9	4.30E-13	110.1
immune response	8	22.9	2.10E-07	17.4
positive regulation of transcription from RNA polymerase II promoter	5	14.3	3.00E-02	4.1
cellular response to starvation	2	5.7	9.60E-02	19.1
regulation of transcription from RNA polymerase II promoter	5	14.3	1.90E-01	2.1

.

Supplementary Table S4. KEGG signaling enrichment analysis of DEGs of adipose tissues between by control and FF fed animal groups by the DAVID Bioinformatics tool.

Term	Count	Percent	P-Value
Osteoclast differentiation	4	1.18E+01	4.30E-03
Phagosome	4	1.18E+01	8.00E-03
Tuberculosis	4	1.18E+01	1.30E-02
Human T-cell leukemia virus 1 infection	4	1.18E+01	1.90E-02
Growth hormone synthesis, secretion and action	3	8.80E+00	3.30E-02
TNF signaling pathway	3	8.80E+00	3.60E-02
Non-alcoholic fatty liver disease	3	8.80E+00	6.00E-02
PI3K-Akt signaling pathway	4	1.18E+01	6.20E-02
Influenza A	3	8.8	7.60E-02
Asthma	2	5.9	9.10E-02

Supplementary Table S5. KEGG signaling enrichment analysis of DEGs of liver tissues between by control and FF fed animal groups by the DAVID Bioinformatics tool.

Term	Count	Percent	P-Value	Fold Enrichment
Allograft rejection	6	14.3	5.40E-07	34.6
Type I diabetes mellitus	6	14.3	6.40E-07	33.5
Graft-versus-host disease	6	14.3	1.00E-06	30.4
Autoimmune thyroid disease	6	14.3	1.70E-06	27.5
Kaposi sarcoma-associated herpesvirus infection	8	19	2.00E-06	12.2
Viral myocarditis	6	14.3	2.40E-06	25.7
Antigen processing and presentation	6	14.3	5.20E-06	22.1
Cellular senescence	7	16.7	5.70E-06	14.2
Epstein-Barr virus infection	7	16.7	4.30E-05	10
Human immunodeficiency virus 1 infection	7	16.7	5.10E-05	9.7
Endocytosis	7	16.7	6.10E-05	9.4
Human cytomegalovirus infection	7	16.7	6.20E-05	9.3
Cell adhesion molecules	6	14.3	1.10E-04	11.7
Phagosome	6	14.3	1.10E-04	11.7
Human T-cell leukemia virus 1 infection	6	14.3	5.10E-04	8.4
Viral carcinogenesis	6	14.3	5.50E-04	8.3
Human papillomavirus infection	6	14.3	2.90E-03	5.7
Herpes simplex virus 1 infection	6	14.3	6.00E-03	4.8
Osteoclast differentiation	4	9.5	7.00E-03	9.7
Fluid shear stress and atherosclerosis	3	7.1	6.50E-02	6.9

Supplementary Table S6. KEGG signaling enrichment analysis of DEGs of muscle tissues between by control and FF fed animal groups by the DAVID Bioinformatics tool.

Term	Count	Percent	P-Value	Fold Enrichment
Human T-cell leukemia virus 1 infection	10	28.6	1.20E-09	17.3
Allograft rejection	7	20	2.70E-09	49.6
Type I diabetes mellitus	7	20	3.30E-09	47.9
Graft-versus-host disease	7	20	6.00E-09	43.6
Autoimmune thyroid disease	7	20	1.10E-08	39.4
Viral myocarditis	7	20	1.70E-08	36.9
Epstein-Barr virus infection	9	25.7	2.80E-08	15.7
Antigen processing and presentation	7	20	4.20E-08	31.6
Human immunodeficiency virus 1 infection	8	22.9	7.60E-07	13.6
Cellular senescence	7	20	1.50E-06	17.4
Cell adhesion molecules	7	20	1.80E-06	16.8
Phagosome	7	20	1.90E-06	16.7
Herpes simplex virus 1 infection	9	25.7	2.30E-06	8.9
Kaposi sarcoma-associated herpesvirus infection	7	20	7.80E-06	13.1
Viral carcinogenesis	7	20	1.30E-05	11.9
Human cytomegalovirus infection	7	20	1.70E-05	11.5
Endocytosis	6	17.1	2.20E-04	9.9
Human papillomavirus infection	6	17.1	1.10E-03	7
Small cell lung cancer	4	11.4	1.30E-03	17.5
Chronic myeloid leukemia	3	8.6	1.40E-02	16
NF-kappa B signaling pathway	3	8.6	2.70E-02	11
Toxoplasmosis	3	8.6	2.80E-02	10.8
MAPK signaling pathway	4	11.4	2.90E-02	5.6
Pathways in cancer	5	14.3	3.40E-02	3.8
Apoptosis	3	8.6	4.30E-02	8.6
Hepatocellular carcinoma	3	8.6	6.10E-02	7.1
Transcriptional m is-regulation in cancer	3	8.6	7.80E-02	6.2

Supplementary Table S7. Community richness and diversity of microbial dynamics in experimental samples.

Groups	16sRNA				
	OTUs	Chao1	Shannon	Gini-Simpson	Good's Coverage
**NF**	458.3 ± 35.0	521.0 ± 35.6	6.366 ± 0.21	0.967 ±0.007	0.993 ±0.001
**AF**	422.3 ± 30.9	484.1 ± 13.9	6.500 ± 0.11	0.974 ±0.002	0.992 ±0.002
			**ITS**		
**NF**	73.6 ±9.01	74.4 ± 9.39	2.23 ±0.34	0.707 ±0.05	0.999 ± 0.00
**AF**	98.3 ± 40.1	101.5 ± 39.0	2.92 ± 0.31	0.808 ± .031	0.999 ± 0.00

OTU: Operational Taxonomic Unit; NF: Normal diet Feed; AF: Alternative diet Feed; ITS: Internal transcribed spacer

Supplementary Table S8. Changes in bacterial communities at phylum level in normal diet feed and alternative diet feed in rumen of *Bos Taurus.*

Phylum	NF diet	AF diet
Archaea;_Euryarchaeota	0.687%	1.189%
Bacteria;_Actinobacteria	0.469%	0.376%
Bacteria;_Bacteroidetes	46.41%	31.19%
Bacteria;_Chloroflexi	0.463%	2.735%
Bacteria;_Elusimicrobia	0.016%	0.014%
Bacteria;_Fibrobacteres	0.173%	0.030%
Bacteria;_Firmicutes	39.53%	42.64%
Bacteria;_Lentisphaerae	0.007%	0.006%
Bacteria;_Planctomycetes	0.272%	0.226%
Bacteria;_Proteobacteria	0.399%	0.287%
Bacteria;_Spirochaetes	0.613%	0.631%
Bacteria;_Synergistetes	0.027%	0.169%
Bacteria;_Tenericutes	0.147%	0.172%
Others	10.77%	20.31%

NF: Normal diet Feed; AF: Alternative diet Feed;

Supplementary Table S9. Changes in bacterial communities at genus level in normal diet feed and alternative diet feed in rumen of *Bos Taurus.*

Genus	NF diet	AF diet
Methanobrevibacter	0.687%	1.189%
Bacteroides	0.960%	1.103%
Barnesiella	7.088%	0.905%
Lentimicrobium	12.987%	5.158%
Duncaniella	6.515%	5.528%
Paludibacter	0.722%	1.433%
Porphyromonas	0.126%	1.370%
Prevotella	14.337%	5.896%
Millionella	0.136%	0.339%
Parabacteroides	0.295%	0.050%
Capnocytophaga	0.644%	2.030%
Galbibacter	0.419%	0.652%
Olivibacter	0.004%	5.026%
Flexilinea	0.463%	2.735%
Limosilactobacillus	0.031%	0.254%
Streptococcus	0.540%	2.159%
Intestinimonas	3.050%	4.820%
Christensenella	1.223%	1.686%
Caminicella	0.077%	0.106%
Ihubacter	0.585%	1.222%
Coprococcus	0.156%	0.055%
Faecalicatena	1.288%	2.091%
Lachnoclostridium	1.069%	1.416%
Novisyntrophococcus	1.619%	0.945%
Pseudobutyrivibrio	0.074%	0.656%
Syntrophococcus	2.779%	1.495%
Ethanoligenens	3.180%	4.303%
Oscillibacter	0.172%	0.578%
Ruminococcus	2.726%	2.315%
Sporobacter	1.578%	0.574%
Succiniclasticum	8.802%	6.363%
Megasphaera	0.006%	0.234%

Supplementary Table S10. Changes in fungal communities at phylum level in normal diet feed and alternative diet feed in rumen of *Bos Taurus.*

Phylum fungi	NF diet	AF diet
Unassigned;Other	0.319%	5.538%
k_Eukaryota_ unidentified	0.015%	0.032%
k_Fungi;Other	0.005%	1.009%
k_Fungi;p_Ascomycota	1.233%	1.065%
k_Fungi;p_Basidiomycota	0.067%	0.187%
k_Fungi;p_Chytridiomycota	0.000%	0.000%
k_Fungi;p_Mucoromycota	0.002%	0.123%
k_Fungi;p_Neocallimastigomycota	98.20%	86.88%
k_Fungi;p_unidentified	0.091%	5.036%
k_Protista;p_unidentified	0.002%	0.000%
k_Viridiplantae;p_Anthophyta	0.060%	0.072%
k_Viridiplantae;p_Chlorophyta	0.005%	0.055%

Supplementary Table S11. Changes in fungal communities at genus level in normal diet feed and alternative diet feed in rumen of *Bos Taurus.*

Genus	NF diet	AF diet
Unassigned	0.319%	5.538%
Talaromyces	0.398%	0.085%
Other	0.398%	0.000%
Nigrospora	0.014%	0.268%
Neocallimastigaceae;Other	4.284%	24.902%
Anaeromyces	0.011%	0.194%
Caecomyces	0.654%	4.894%
Neocallimastix	72.022%	25.201%
Orpinomyces	21.112%	29.703%
Piromyces	0.117%	1.851%
unidentified	0.091%	5.036%
Nannochloris	0.001%	0.000%
